# A review of computed tomography patterns of metastatic breast cancer patients undergoing treatment at a private oncology centre in Ghana

**DOI:** 10.11604/pamj.2021.38.50.21945

**Published:** 2021-01-18

**Authors:** Yaw Boateng Mensah, Clement Edusa, Josephine Nsaful, Naa Adjeley Mensah

**Affiliations:** 1 Department of Radiology, University of Ghana Medical School, College of Health Sciences, Korle Bu, Accra, Ghana,; 2 Sweden Ghana Medical Centre, Accra, Ghana,; 3 Department of Surgery, University of Ghana Medical School, College of Health Sciences, Korle Bu, Accra, Ghana,; 4 Regional Institute of Population Studies, University of Ghana, Legon, Accra, Ghana

**Keywords:** Breast cancer, metastasis, computed tomography, Ghana, histological subtypes

## Abstract

**Introduction:**

breast cancer is the commonest malignant disease in Ghanaian women and accounts for 17% of cancer-related deaths in the country. It has been classified into molecular subtypes depending on the presence or absence of hormone receptors and the human epidermal growth factor receptor 2. Computed tomography is often the preferred modality for monitoring metastatic disease due to its ability to determine the extent of local and metastatic disease.

**Methods:**

this was a retrospective study conducted at Sweden Ghana Medical Centre (SGMC). Hospital records and chest and abdominal computed tomography (CT) scan images of breast cancer patients who had been managed at SGMC between June 2016 and August 2019 were used to document age, gender, histological group, type of surgical intervention done, molecular subtypes of the disease and imaging findings. Microsoft Excel 2016 and SPSS version 20.0 were used to summarise the data obtained into tables, charts and to test for significant associations.

**Results:**

the most common site of breast cancer metastasis was lymph nodes. The three commonest sites of distant metastases were the lung seen in 44 patients (55.3%), bone in 37 patients (44.6%) and liver in 33 patients (39.8%). Chi square test for association between the molecular subtypes of the breast cancer and proportion of patients that showed a particular type of metastases revealed that, the differences noted for lung, pleural and cardiac metastases were statistically significant, that for bone and liver were not.

**Conclusion:**

breast cancer commonly metastasised to lymph nodes, lung, bone, liver, pleura and heart in descending order. The commonest CT patterns for metastases were multiple nodules for lung, effusion for pleura and heart and osteolytic lesions for bone.

## Introduction

Breast cancer is currently the commonest malignant disease in Ghanaian women and accounts for about 17.1% of the cancer- related deaths in the country [1, 2]. This disease is the second most common cause of cancer-related deaths in the Western world [1, 3]. Breast cancer is classified into histological subtypes using hormone receptors namely oestrogen receptor (ER) and progesterone receptor (PR) as well as human epidermal growth factor receptor 2 (HER2). The subtypes are luminal A (ER/PR positive, HER2 negative, low Ki-67 level); Luminal B (ER/PR positive, HER2 positive/HER2 negative with high Ki-67 level, triple negative/basal-like (ER/PR negative, HER2 negative); HER2-enriched (ER/PR negative, HER2 positive); normal-like which has similar hormone status as luminal A but has a slightly worse prognosis than luminal A [3, 4]. Radiology plays a significant role in breast cancer management viz early detection with screening mammography and ultrasound, staging and surveillance for recurrence after the initial treatment [3, 5, 6].

Computed tomography (CT), magnetic resonance imaging (MRI), and bone scintigraphy are used in the management of advanced disease [6]. Multidetector CT scan is preferred for monitoring metastatic breast cancer due to high accuracy in determining extent of local disease, demonstrating metastasis and treatment complications [7]. Breast cancer studies in Ghana have centred on clinical features, patient attitudes, evaluation of pathological samples and molecular studies of the samples [1, 8-10]. Little scientific data is available on imaging findings in breast cancer patients in Ghana even though radiology plays a major role in the diagnosis and management of the disease. Documenting imaging findings in breast cancer patients adds value to scientific knowledge, is an adjunct to clinical finding, direct treatment options and audit both clinical and pathological decisions. This study aims to document the common thoracic and abdominal CT scan patterns of patients with metastatic breast cancer and ascertain the relationship between molecular sub-type and extent of disease.

## Methods

This was a retrospective study conducted at Sweden Ghana Medical Centre (SGMC), a private facility that provides complete clinical oncology care in Ghana. The centre sees patients with all kinds of cancer from countries in the West African sub region. Clinical information of the breast cancer patients who were managed at SGMC between June 2016 and August 2019 and their CT scan images was used for the study.

Breast cancer patients managed at SGMC and had at least one chest and abdominal CT during treatment were included in the study. Information documented included age, gender, histological group and molecular subtypes of the disease. Thoracic and abdominal CT scan images of the patients were reviewed by a senior radiologist and significant findings documented. Information was collected on an MS excel spreadsheet and analysed using SPSS 20 in a password protected computer.

Data on age, gender, histological group and molecular subtype of the disease and the CT scan findings were analysed using descriptive statistics.

Test of association (Pearson´s Chi-square) was conducted between the molecular subtypes of the disease and the CT scan findings of the patients.

### Ethical issues

Ethical approval was obtained from the Institutional Review Board of Korle Bu Teaching Hospital. Confidentiality of patient information obtained from the hospital records and the imaging findings was ensured. Permission was also sought from the management of SGMC to use the information for this study.

## Results

**Figure 1:**
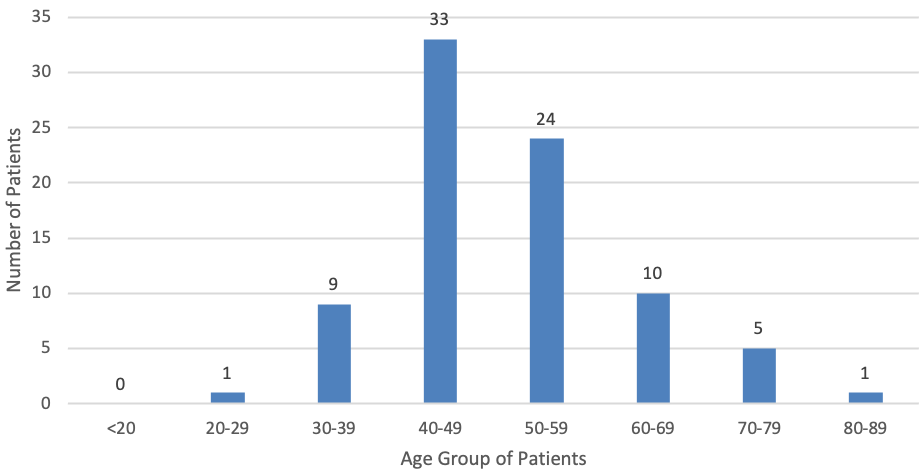
age distribution of patients

One hundred and ninety (190) CT scan studies for 83 patients were evaluated out of which 82 (98.7%) were females and one was a male (1.3%). The mean age of the patients was 51.2 years with a standard deviation of 10.9 years and a median age of 49.0 years. The youngest patient was 29 years and the oldest 80 years. The commonest age group was the 40-49 years, followed by 50-59 years and 60-69 years as shown in Figure 1.

Forty-nine patients (59%) did not have metastasis at the time of diagnosis while 34 patients (41%) had. Information on histological subtypes was available for 76 patients (91.6%). These were invasive carcinoma No Special Type (NST) 66 patients (86.8%), invasive lobular carcinoma 3 patients (3.9%), poorly differentiated carcinoma, 2 patients (2.6%) and mammary adenocarcinoma, 2 patients (2.6%). The rest were mammary Paget´s disease, medullary carcinoma and papillary carcinoma, each seen in 1 patient (1.3%).

Information on the molecular subtypes was available for 67 patients (80.7%) in the medical records. Of these, 22 patients (32.8%) were luminal A/ normal-like, 21 patients (31.3%) were triple negative, 16 patients (23.9%) were luminal B and 8 patients (11.9%) were HER 2-enriched. The information available made it difficult to separate luminal A from normal-like patients.

**Figure 2:**
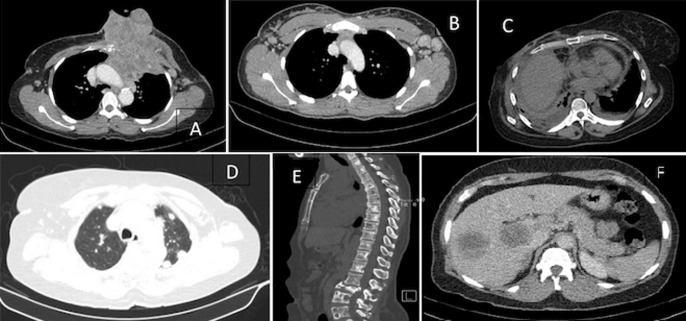
axial computed tomography scan images of various patterns of breast cancer metastases: A) direct extension of the disease to the anterior mediastinum; B) lymph node metastasis-bilateral enlarged and infiltrated axillary lymph nodes; C) bilateral malignant pleural effusion and pericardial effusion; D) multiple metastatic lung nodules; E) osteolytic, osteoblastic and mixed bone metastatic patterns; F) liver metastasis - hypodense nodules which enhance less than normal liver parenchyma

Sixty-four patients (77.1%) had at least one lymph node group involvement (Figure 2). The four most common groups were the axillary, 60 patients, (66.7%), mediastinal 10 patients (11.1%), supraclavicular, 7 patients (7.8%) and internal mammary 6 patients (6.7%). Fifty-four patients (65.1%) had lung metastases (Figure 2). Features included nodular pattern in 43 patients (61.5%), consolidation in 14 patients (19.2%), lymphangitic spread in 12 patients (16.4%) and endobronchial spread in 2 patients (2.7%). Nineteen patients showed more than one pattern. Twenty-five patients (30.1%) showed evidence of pleural metastases with pleural effusion (Figure 2) being the commonest pattern, 20 patients (71%). Eight patients (9.6%) showed evidence of cardiac metastasis with pericardial effusion (Figure 2) being the commonest pattern, 5 patients (56%).

Thirty-seven patients (44.6%) showed bone metastasis (Figure 2) with 95.5% of them showing lesions in multiple sites. Sixteen patients (43%) had osteolytic pattern, 14 (38%) had mixed osteolytic-osteoblastic pattern and 7 (19%) had osteoblastic pattern. The sites of bone metastasis were the vertebral column, 30 patients (81.1%), pelvic girdle, 18 patients (48.6%), sternum, 14 patients (37.8%), shoulder girdle, 13 patients (35.1%), upper limb, 9 patients (24.3%), and lower limb, 5 patients (13.5%). Thirty-three patients (39.8%) had liver metastasis (Figure 2) with all but three of the patients (9.1%) showing metastases in other organs.

Table 1 shows the number of each patients with a particular molecular subtype that spread to the different metastatic sites. Pearson's Chi square test for associations showed a significant statistical difference between the patients´ molecular subtype and the proportion of patients with lung (X^2^(1,N = 67) = 5.388, p = 0.020), pleura (X^2^(1, N = 67) = 12.552, p < 0.001), lymph node (X^2^(1, N = 67) = 201.555, p < 0.001) and cardiac metastases (X^2^(1, N = 67) = 11.879, p = 0.001). No such association was noted in those with bone (X^2^(1, N = 67) = 2.522, p = 0.112) and liver (X^2^(1, N = 67) = 1.806, p = 0.179) metastasis.

**Table 1:** molecular subtypes of breast cancer and metastatic site

Molecular subtypes	Metastatic Site																
	Lung			Bone			Liver				Pleural				Cardiac		
	Yes	No	Total	Yes	No	Total	Yes	No	Total	Yes		No	Total	Yes		No	Total
**Luminal A/ Normal-like**	13	9	22	11	11	22	10	12	22	5		17	22	5		17	22
**Luminal B**	13	3	16	8	8	16	6	10	16	4		12	16	4		12	16
**HER2-enrched**	5	3	8	4	4	8	5	3	8	2		6	8	2		6	8
**Triple Negative**	12	9	21	4	17	21	7	14	21	8		13	21	8		13	21
**Total**	43	24	67	27	40	67	28	39	67	19		48	67	19		48	67

## Discussion

The mean age for patients in the study was 51.2 years, SD-10.9 years. This is higher than the 43-46 years reported in studies by Clegg-Lamptey *et al*. Ohene-Yeboah and Adjei, and Mensah *et al*. reported in 2009, 2012 and 2016 respectively [1, 8, 9]. It is believed that some patients live a little longer before developing the metastasis hence are slightly older than the average age of general breast cancer population. Again, there is also a possibility that there has been improvement in survival rate since the last study was published 3 years ago. The fact that 59% of the patients did not have metastasis at presentation corroborates this.

The highest number of patients in this study were in the 40-49 year-group as was also reported by Ohene-Yeboah and Adjei in Kumasi, Ghana [9]. In their study, the second largest group was the 30-39 year group while this study found the second and third largest groups to be the 50-59 and 60-69 year groups with the fourth largest being the 30-39 year group. This further supports the suggestion that the patients in this study may have survived long enough to develop metastases, started with less aggressive disease or possibly improved treatment mentioned in the previous paragraph.

Invasive carcinoma NST was the highest histologic type in the patient population. It was noted in 66 patients (86.8%). This compares with work done by Edmund *et al*. in KBTH in 2013 which found 91.9% of breast cancers to be invasive ductal carcinoma. This was followed by invasive lobular carcinoma similar to what was noted in this study [11]. The three commonest histological subgroups in this study were luminal A/normal-like 32.8%, triple negative 31.3% and luminal B 23.9%. Mensah *et al*. documented the same top three, but reported triple negative 66%, luminal A, 19% and HER2-rich, 9.5% [1]. This could be because we studied only metastatic disease, while they studied all breast cancer patients. Again, triple negative subtype is associated with a poorer prognosis than luminal A, thus the latter group may live longer and possibly outnumber the former as the years go by [1].

Seventy-one percent of the patients had significant lymph node involvement, with 76.5% of these involving more than one lymph node group. This is not unexpected as lymphatic dissemination is the most prominent mode of breast cancer spread as has been reported by Thomas *et al*. [12] That explains why lymph node involvement was relatively far more than the proportions of distant metastasis noted in this study. Lung metastasis was second to lymph node metastasis. Like other studies, the CT patterns of lung metastasis were nodular, consolidation, lymphangitic and endobronchial with nodular being the commonest (57.4%) [7, 13]. Thomas *et al*. in their autopsy series that found lymphangitic spread as the commonest pattern. The difference in findings has been explained by the limited sensitivity of CT scan in identifying lymphatic spread of breast cancer [12]. The same reason can be adduced for why autopsy study found 71% pleural metastasis while this study reported only 30%. Cardiac spread was noted in 8% of the patients which is similar to 5-18% noted by Jung JI *et al*. [13]. Breast cancer is the commonest extra thoracic cancer that spreads to the heart [13].

Bone metastasis seen in 45% of the patients agrees with several studies [13]. Three patterns of bone metastases were noted on CT scan, i.e. osteolytic, 43%, osteoblastic, 19% and mixed (38%). Other studies found similar trends and attributes the mixed type to the osteolytic type which is responding to treatment [13]. This study like others also identified the spine as the commonest site of bone metastases (81.1%) [13].

Liver metastasis was noted in 40% of the patients comparable to 50% reported by Michaels *et al*. [7]. CT imaging protocol plays a significant role in detection of metastasis and could be the reason why the ratios are different. Only 9% of liver metastases patients had isolated metastases; the rest had metastases in other parts of the body which may suggesting liver metastasis is a sign of extensive disease.

This study found a significant association between the molecular subtypes and the proportion of patients with lung, pleural, lymph node and cardiac metastasis, but not with liver and bone metastasis. This supports studies that suggest that some molecular subtypes have unique characteristics that influence the behaviour of the disease [3]. Triple negative and HER2 subtypes are known to be associated with increased metastatic rate [14] and low survival outcomes [1].

**Limitations:** the retrospective nature of the study made it difficult to elicit information that was not available in the patient´s record which may have affected important proportions in the study.

## Conclusion

The common sites of breast cancer metastasis in our patients were lymph nodes, lung, bone, liver, pleura and heart in descending order. The commonest CT metastatic patterns were, nodular for lung metastasis, effusion for pleura and cardiac effusion, osteolytic for bone. There was a significant association between the molecular subtypes and the proportion of patients with lung, pleural, cardiac, lymph node group involvement but not bone and liver. Further studies should be done to understand the behavior of breast cancer in the Ghanaian population in order to improve management of the disease. There is a need for more funding in breast cancer management and research to improve clinical outcomes of the disease.

### What is known about this topic

- Breast cancer metastasises to lymph node, lung, bone and liver in descending order;
- The three commonest histological subgroups in breast cancer patients in Africa are luminal A/normal-like, triple negative and luminal B.

### What this study adds

- Liver metastasis in breast cancer is a sign of grave disease with poor prognosis;
- Information being provided is that of indigenous African subjects not African Americans especially as the situation persists in the West African subregion.
